# Glycosaminoglycans affect endothelial to mesenchymal transformation, proliferation, and calcification in a 3D model of aortic valve disease

**DOI:** 10.3389/fcvm.2022.975732

**Published:** 2022-09-29

**Authors:** Jonathan Alejandro Bramsen, Bridget R. Alber, Melissa Mendoza, Bruce T. Murray, Mei-Hsiu Chen, Peter Huang, Gretchen J. Mahler

**Affiliations:** ^1^Department of Biomedical Engineering, Binghamton University, Binghamton, NY, United States; ^2^Department of Biomedical Engineering, George Washington University, Washington, DC, United States; ^3^Department of Mechanical Engineering, Binghamton University, Binghamton, NY, United States; ^4^Department of Mathematics and Statistics, Binghamton University, Binghamton, NY, United States

**Keywords:** calcific aortic valve disease, chondroitin sulfate, hyaluronic acid, mechanobiology, fibrosa layer

## Abstract

Calcific nodules form in the fibrosa layer of the aortic valve in calcific aortic valve disease (CAVD). Glycosaminoglycans (GAGs), which are normally found in the valve spongiosa, are located local to calcific nodules. Previous work suggests that GAGs induce endothelial to mesenchymal transformation (EndMT), a phenomenon described by endothelial cells’ loss of the endothelial markers, gaining of migratory properties, and expression of mesenchymal markers such as alpha smooth muscle actin (α-SMA). EndMT is known to play roles in valvulogenesis and may provide a source of activated fibroblast with a potential role in CAVD progression. In this study, a 3D collagen hydrogel co-culture model of the aortic valve fibrosa was created to study the role of EndMT-derived activated valvular interstitial cell behavior in CAVD progression. Porcine aortic valve interstitial cells (PAVIC) and porcine aortic valve endothelial cells (PAVEC) were cultured within collagen I hydrogels containing the GAGs chondroitin sulfate (CS) or hyaluronic acid (HA). The model was used to study alkaline phosphatase (ALP) enzyme activity, cellular proliferation and matrix invasion, protein expression, and calcific nodule formation of the resident cell populations. CS and HA were found to alter ALP activity and increase cell proliferation. CS increased the formation of calcified nodules without the addition of osteogenic culture medium. This model has applications in the improvement of bioprosthetic valves by making replacements more micro-compositionally dynamic, as well as providing a platform for testing new pharmaceutical treatments of CAVD.

## Introduction

Calcific aortic valve disease (CAVD) is characterized as a complex and multi-factorial progression involving altered matrix organization and dysregulated crosstalk between resident cell populations of the aortic microenvironment, ultimately leading to a disrupted tri-layer organization of the aortic valve, the presence of calcium deposits, and inhibited valve function (1). This multi-step cascade of events results in an estimated 130,000 deaths annually in the USA (2). Valve replacement surgery is currently the only reliable treatment option, and these inert replacements lack the ability to biologically integrate and function long-term. Tissue engineering is a promising approach to help decrease the societal burden of CAVD by creating biologically inspired and mimetic valve replacements. Developing precise models of healthy and diseased valves could accelerate the transition of tissue engineered valves from bench to bedside. This study developed a platform for studying the aortic valve microenvironment to better understand matrix remodeling and how it plays a role in matrix mineralization. The platform can have applications in the improvement of bioprosthetic valves by making replacements more micro-compositionally dynamic, thereby addressing a major limitation in current tissue engineered constructs, as well as providing a platform for testing new pharmaceutical treatments of CAVD.

Valve interstitial cells (VICs) are critical to the function and overall homeostasis of the aortic valve (3). VICs and their extracellular matrix (ECM) proteins are known to play a role in both the early stages of valvulogenesis (4–6) and in aortic valve disease progression (7, 8). The adult aortic valve has three semilunar leaflets, each characterized by distinct ECM. The fibrosa layer is composed of circumferentially aligned collagen I fibers, the spongiosa is composed of collagen and randomly oriented proteoglycans rich in glycosaminoglycans (GAGs), and the ventricularis is composed of primarily radially orientated elastin (9). Early aortic valve disease includes ECM remodeling, cell proliferation, and increased alkaline phosphatase (ALP) activity (10–13). ALP promotes calcification by reducing pyrophosphate and osteopontin *via* hydrolysis (14). Late-stage aortic valve disease is characterized by the presence of calcific nodules on the aortic valve fibrosa and a disruption of the valvular ECM organization (15). Specifically, GAGs have been shown to relocate to the fibrosa layer in diseased valves and have been implicated in facilitating disease progression due to their localization near calcific nodules (16–18).

Endothelial to mesenchymal transformation (EndMT) is a phenomenon described by endothelial cells’ loss of endothelial markers such as platelet endothelial cell adhesion molecule-1 (PECAM-1), gaining of mesenchymal cell markers such as alpha smooth muscle actin (α-SMA), and gaining of migratory properties (19). EndMT plays a major role in the early stages of valve development (20), has been implicated in valvular pathology, and may serve as a mechanism to replenish adult interstitial cell populations (21). Previous work has shown that GAGs induce EndMT in an *in vitro* porcine cell model, and mesenchymally transformed cells and GAGs are found near calcified nodules in diseased human valves (17). Later studies showed that GAGs also promote calcification in an *in vitro* model with porcine cells (22). In the current study, the 3D model with porcine aortic valve cells simulated the aortic valve fibrosa layer and was used to study the role of EndMT-derived activated valve interstitial cells (aVICs) on the progression of CAVD. Although 3D models have been applied elsewhere (23–26), some incorporating GAGs (27–30), questions remain on the role of the ECM on aVIC behavior and how these EndMT-derived cells may contribute to the onset and progression of matrix mineralization (31). Here, a 3D collagen I hydrogel scaffold containing chondroitin sulfate (CS) or hyaluronic acid (HA) and seeded with porcine aortic valve endothelial and interstitial cells was used to study EndMT-derived aVICs by quantifying: (1) ALP activity, (2) calcified nodule formation *via* Alizarin Red S (ARS) staining, and (3) cell phenotype and protein expression with immunocytochemistry, flow cytometry, cellular invasion, and proliferation assays.

## Materials and methods

### Cell isolation and culture

Porcine aortic valve cell isolation and culture have previously been described (17, 32). Briefly, porcine aortic valve interstitial cells (PAVIC) and porcine aortic valve endothelial cells (PAVEC) were obtained from freshly slaughtered pigs at a local abattoir. Cells were pooled from young (6–8 months) females or castrated males. Following isolation *via* collagenase digestion (600 U/mL collagenase type II, Worthington Biochemical Corporation, Lakewood, NJ, USA) PAVIC were grown in Dulbecco’s Modified Eagle Medium (DMEM, Invitrogen, Waltham, MA, USA) supplemented with 1% penicillin-streptomycin (Pen-Strep, Invitrogen) and 10% fetal bovine serum (FBS, VWR, Radnor, PA, USA). PAVEC were grown on 50 μg/mL rat tail collagen I (Corning Life Sciences, Tewksbury, MA, USA)-coated flasks and in medium supplemented with 50 U/mL heparin sulfate (Sigma-Aldrich, St. Louis MO, USA). PAVIC and PAVEC were used between passages 3 and 5. During experiments, medium was changed every 48 h with the PAVIC formulation.

### 3D hydrogel preparation

A 3D collagen hydrogel co-culture model of the aortic valve microarchitecture was created to recapitulate late-stage disease conditions (i.e., GAG infiltration of the fibrosa layer) *in vivo*. The hydrogels were formed by seeding PAVIC into and PAVEC on top of the hydrogels. Briefly, PAVIC (1 × 10^6^ cells/mL) were suspended within a solution containing ice-cold 3× DMEM, 18 MΩ water, FBS, 0.1 M sodium hydroxide (NaOH), and rat tail collagen I. Then, 300 μL was pipetted into a 24-well plate (Corning) and allowed to crosslink at 37°C for 1 h. Following incubation, PAVEC (95,000 cells/cm^2^) were added on top of hydrogel constructs in PAVIC medium. Four conditions were used: 1.5 mg/mL collagen (control), 2.2 mg/mL collagen (stiffness control), 1.5 mg/mL collagen + 20 mg/mL CS (chondroitin sulfate A sodium salt from bovine trachea, Sigma-Aldrich), and 1.5 mg/mL collagen + 20 mg/mL HA (hyaluronic acid sodium salt from *Streptococcus equi*, Sigma-Aldrich). Previous work showed that 2.2 mg/mL collagen gels can serve as stiffness controls for 1.5 mg/mL collagen gels with 20 mg/mL GAG-supplementation (17). All cultures were incubated at 37°C and 5% CO_2_ for 2 weeks before analysis. Cells were cultured for 14 days to mimic previous relevant studies, which demonstrated significant calcific nodule formation while also maintaining cell viability in 14 day valve cell co-cultures (22, 31, 33).

### Alizarin Red S assay

Alizarin Red S (ARS) (Sigma-Aldrich) was used to quantify calcific nodule formation (33, 34). Briefly, hydrogels were washed with 1× phosphate buffered saline (PBS, Omnipur^®^, Baltimore, MD, USA) and fixed with 4% paraformaldehyde (PFA, Sigma-Aldrich) overnight at 4°C. A 40 mM ARS stain solution was added, and then rinsed with 1× PBS. Bright field images were taken using a Nikon Eclipse Ts2 at 20× magnification to visualize ARS-stained area. After imaging, a 10% acetic acid solution was used to release ARS and a 10% ammonium hydroxide solution was used to neutralize. Absorbance was measured with a plate reader at 405 nm and concentration was calculated using a standard curve (35).

### Alkaline phosphatase assay

Alkaline phosphatase was used to investigate early stage valve disease progression. After 2 weeks of growth, gels were enzymatically digested with a 600 U/mL collagenase type II solution (Worthington Biochemical Corporation). Once cells were in solution, they were rinsed with 1× PBS *via* centrifugation, resuspended in sterile 18 MΩ water, and sonicated for 20 min. An ALP substrate (pNPP, Sigma-Aldrich) was then added to each cell lysate, and read at 405 nm using a plate reader, and concentration was calculated using a standard curve of p-nitrophenol (Sigma-Aldrich). P-nitrophenol values were normalized to total protein content in hydrogels obtained with Bradford assays (Sigma-Aldrich).

### Immunocytochemistry

Immunocytochemistry techniques were used to co-label α-SMA and PECAM-1. A detailed protocol was described by Dahal et al. (17). Following 2 weeks of growth, hydrogels were rinsed with 1× PBS and fixed overnight at 4°C with 4% PFA. Samples were then permeabilized with 0.2% Triton X-100 (Sigma-Aldrich) and blocked with 1% bovine serum albumin (BSA, Rockland™, Limerick, PA, USA) diluted in 1× PBS overnight at 4°C. Primary antibodies for α-SMA (Abcam ab125044) at a 1:100 dilution and for PECAM-1 (P2B1, DHSB) at 2 μg/mL were both added in 1× PBS and incubated overnight at 4°C. Dilutions of 1:1000 of secondary antibodies for α-SMA [Goat Anti-Rabbit IgG H&L Alexa Fluor^®^ 488 (ab150077)] and PECAM-1 [Goat Anti-Mouse IgG H&L Alexa Fluor^®^ 568 (A11008)] were added. Samples were incubated for 2 h at room temperature (RT) and protected from light. Secondary antibodies were rinsed and DRAQ-5 (ThermoFisher Scientific, 62251) was added at a dilution of 1:1000 and incubated for 30 min at RT and protected from light. Hydrogels were then imaged using a Zeiss LSM 880 Two-Photon confocal microscope. An image analysis code was implemented in MATLAB to compute the amount of α-SMA expression relative to DNA content.

### Fluorescence activated cell sorting

Flow cytometry-based FACS (fluorescence-activated cell sorting) was used to quantitatively evaluate resident cell populations. Hydrogels were rinsed with 1× PBS and digested using 400 μL of a 600 U/mL collagenase solution for 1–2 h, with agitation every 30 min. Once the cells were free from the matrix, the solution was centrifuged at 106^°^ × *g* for 5 min and the cells were rinsed once with 1× PBS and then resuspended in FACS buffer (25 mM HEPES + 2 mM EDTA + 2% FBS in 1× PBS). The cell suspension for each condition was then transferred into a well of a U-bottom 96-well plate, centrifuged at 106^°^ × *g*, resuspended in 100 μL of primary antibody for extracellular marker PECAM-1 (P2B1, DHSB) at 2 μg/mL, and incubated on ice for 30 min. Samples were then centrifuged at 106^°^ × *g*, resuspended in 1:100 of secondary antibody Goat-Anti-Mouse IgG H&L Alexa Fluor^®^ 647 (ab150115), and incubated on ice for 30 min before centrifugation. The cell solution was then fixed and permeabilized by incubating the suspension in BD Biosciences Fix/Perm buffer (554714) for 20 min at RT and protected from light. The buffer was then removed, samples were resuspended in 100 μL of primary antibody for intracellular marker α-SMA (ab125044) at a 1:50 dilution, and incubated for 30 min on ice. The samples were then centrifuged at 106^°^ × *g*, resuspended in 1:100 of secondary antibody Goat Anti-Rabbit IgG H&L Alexa Fluor^®^ 488 (ab150077), and incubated on ice for 30 min before centrifugation and resuspension in FACS buffer for analysis. Negative controls with only secondary antibody stains were also evaluated to quantify both cell autofluorescence and non-specific binding of the secondary antibodies. Solutions were processed with the BD FACS Aria II flow cytometer and analyzed using FlowJo v10 software.

### CellTrace™ analysis

To track generational activity of resident cell populations, CellTrace™ (ThermoFisher Scientific, Waltham, MA, USA) proliferation kits were used to stain PAVICs and PAVECs individually. Following the manufacturer’s protocol, after trypsinizing, PAVICs were resuspended in 1× PBS and then incubated with the CellTrace™ CFSE Proliferation Kit (ThermoFisher C34554) stock solution diluted in DMSO (Invitrogen) for 20 min at 37°C, protected from light. Culture media was then added and incubated for 5 min before removing free dye from the solution. The PAVIC suspension was then centrifuged and resuspended in ice-cold 3× DMEM, 18 MΩ water, and FBS before adding NaOH and collagen I to form hydrogels. Gels were incubated for 1 h at 37°C to crosslink. The staining process was repeated for PAVECs using the CellTrace™ Far Red Proliferation Kit (Thermofisher C34564). PAVECs were then resuspended in warm PAVIC medium before being plated on top of hydrogels. Gels were grown for 2 weeks and either imaged intact (Zeiss LSM 880) or digested with a 600 U/mL collagenase type II solution to extract cells and rinsed with 1× PBS, before analyzing with the BD FACS Aria II flow cytometer. Proliferation activity, also referred to as generational activity, was assessed by quantifying cell divisions of PAVICs and PAVECs using the FlowJo proliferation modeling tool. Z-stack confocal images of CellTrace™-stained intact hydrogels were taken and cellular invasion activity was quantified in MATLAB, thereby probing resident cell and matrix activity.

### Data processing and statistical analysis

All data were processed, analyzed, and graphed in GraphPad Prism 9. Non-parametric Kruskal–Wallis tests with Dunn’s *post hoc* multiple comparisons tests were used to test differences among all experimental groups due to small sample sizes. Sharpiro–Wilks tests were used to verify the normality assumptions. The Spearman’s correlation coefficient was used to measure the association between the number of cells invaded and the average distances traveled when the normality assumptions were violated. A *p*-value < 0.05 was considered statistically significant. Experimental sample sizes (n) were specified in figure legends.

## Results

### Disease progression analysis

**FIGURE 1:**
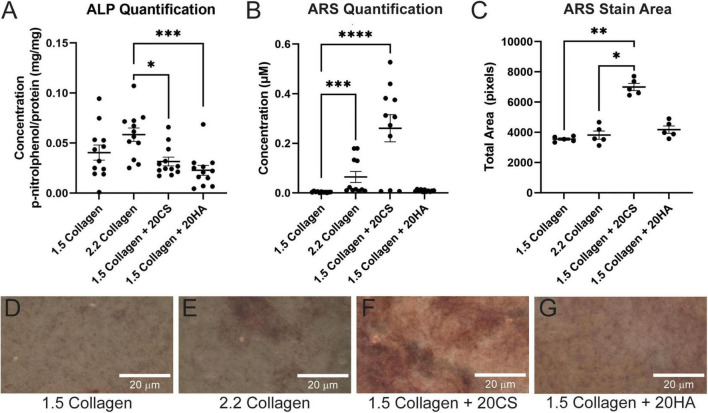
Chondroitin sulfate promotes calcific nodule formation and glycosaminoglycans (GAGs) do not produce increased alkaline phosphatase (ALP) activity. **(A)** ALP quantification in mg p-nitrophenol per mg protein (mg/mg) of digested hydrogels. *n = 12*. **(B)** Quantification of ARS-stained digested hydrogel samples. *n* = 11. **(C)** ARS stain area processed with ImageJ. *n = 5*. Data represented as mean ± SEM. **(D–G)** Brightfield images of intact ARS-stained hydrogels using a Nikon Eclipse Ts2 at 20×. Statistical significance was determined with a non-parametric Kruskal–Wallis test with Dunn’s *post-hoc* test. *p < 0.05, **p < 0.01, ***p < 0.001, and ****p < 0.0001. Scale bars = 20 μm.

Following 14 days in culture, samples were analyzed for both early stage ALP activity and late-stage calcification with ARS assays. All conditions demonstrated ALP activity less than 0.11 mg p-nitrophenol/mg protein, and GAGs conditions showed no significant difference in ALP activity when compared to the 1.5 mg/mL collagen-only controls. The 2.2 mg/mL collagen stiffness control was significantly higher than both experimental GAG conditions, suggesting that stiffness contributed more significantly to early stage disease than GAG presence (Figure 1A).

Alizarin Red S staining was used to locate calcific nodule formation in hydrogels. Intact hydrogels stained with ARS were imaged (Figures 1D–G) and analyzed using ImageJ. ARS stain quantification with a plate reader showed that an increase in stiffness (2.2 mg/mL collagen) induced calcification, and 20 mg/mL CS further significantly induced matrix mineralization. However, HA did not exhibit the same effect (Figure 1B). Stain quantification results were consistent with those from the quantification of the stained area in ARS images (Figure 1C).

### Protein expression

**FIGURE 2:**
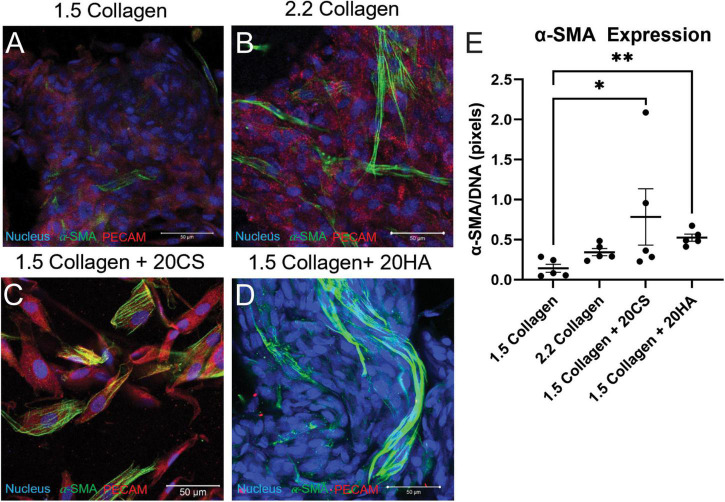
Glycosaminoglycans (GAGs) conditions express higher α-SMA per unit DNA than controls. **(A–D)** Confocal images of intact hydrogels co-stained for α-SMA (green), PECAM-1 (red), and DNA (blue) after 14 days of growth. Scale bars = 50 μm. **(E)** α-SMA expression quantified using a MATLAB script, represented as pixels of α-SMA (green signal) per pixels DNA (blue signal). Data represented as mean ± SEM. Statistical significance was determined with a non-parametric Kruskal–Wallis test with Dunn’s *post-hoc* test. *p < 0.05 and **p < 0.01. *n = 5*.

Hydrogels were co-stained for PECAM-1 (endothelial marker) and α-SMA (activated fibroblast marker) to determine protein expression and cell transformation after 14 days. Confocal images (Figures 2A–D) showed an increase in α-SMA expression in conditions containing the GAGs CS and HA. A semi-quantitative analysis of α-SMA expression in MATLAB verified a significant increase of α-SMA expression in GAGs conditions compared to 1.5 mg/mL collagen controls (Figure 2E).

**FIGURE 3:**
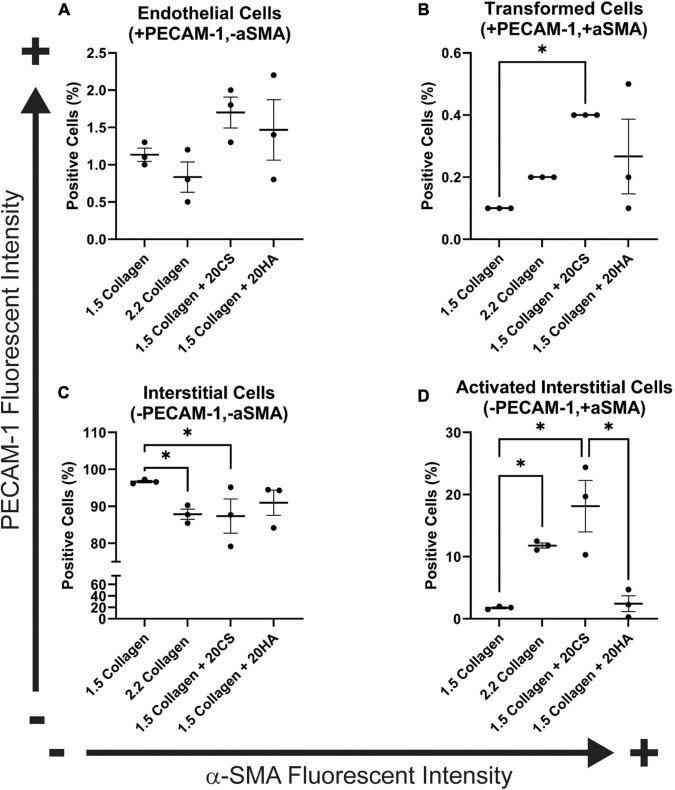
Flow cytometry analysis of protein expression shows an increase in transformed cells in glycosaminoglycans (GAGs) conditions, and an increase in activated interstitial cells in the presence of chondroitin sulfate (CS). Following 14 days of growth, hydrogels were degraded, and isolated cells were processed using a BD FACS Aria II flow cytometer. Cells were stained with α-SMA (Alexa Fluor^®^ 488) and PECAM-1 (Alexa Fluor^®^ 647). **(A)** Endothelial cells (positive for PECAM-1 and negative for α-SMA). **(B)** Cells that have undergone transformation (positive for both PECAM-1 and α-SMA). **(C)** Interstitial cells (negative for PECAM-1 and α-SMA) and **(D)** Activated interstitial cells (negative for PECAM-1 and positive for a-SMA). Data represented as mean ± SEM. Statistical significance was determined with a non-parametric Kruskal–Wallis test with Dunn’s *post-hoc* test. *p < 0.05. *n* = 3.

To quantify α-SMA and PECAM-1 expression, hydrogels were digested, immunocytochemistry staining was used, and cells were analyzed with flow cytometry using a standardized gating procedure in the FlowJo software. Across the four conditions, the percentage of PAVECs (+PECAM-1, −α-SMA) remained consistent (Figure 3A). The number of transformed cells (+PECAM-1, +α-SMA) increased in the presence of CS (Figure 3B). The number of PAVICs (−PECAM-1, −α-SMA) decreased in the presence of CS when compared to controls. The 2.2 mg/mL collagen stiffness control condition also had a lower number of PAVICs when compared to 1.5 mg/mL collagen condition (Figure 3C). CS conditions resulted in a higher percentage of aVICs (−PECAM-1, +α-SMA) when compared to HA and 1.5 mg/mL collagen conditions. Similarly, the 2.2 mg/mL collagen stiffness controls also had a significant increase in aVICs when compared to 1.5 mg/mL collagen controls (Figure 3D). Results from the confocal images and semi-quantitative analysis of relative α-SMA expression were similar, both indicating increases in α-SMA expression in the presence of CS (Figures 2E, 3B,D).

### Cellular invasion

**FIGURE 4:**
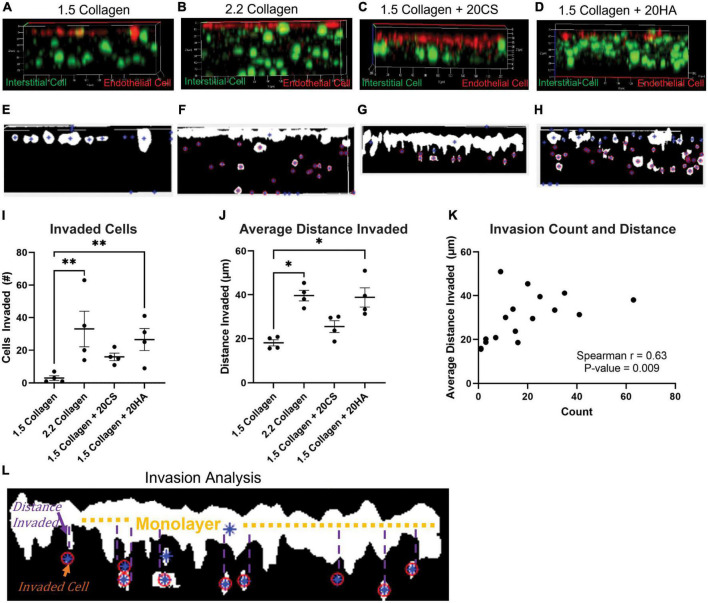
Glycosaminoglycans (GAGs) conditions promote cellular invasion. **(A–D)** Z-stack confocal images taken with a Zeiss LSM 880 Two-Photon confocal microscope. Interstitial cells were stained with CellTrace CSFE-Green (green) and endothelial cells with CellTrace Far-Red (red) prior to hydrogel seeding. Red cells that have migrated from the cell monolayer are classified as invaded. **(E–H)** Examples of MATLAB processed z-stack images used to quantify **(I)** the number of invaded cells and **(J)** the average distance each cell travelled. Data represented as mean ± SEM. *n* = 4. Statistical significance was determined with a non-parametric Kruskal–Wallis test with Dunn’s *post hoc* test. *p < 0.05, ^**^p < 0.01, ^***^p < 0.001, ^****^p < 0.0001. **(K)** Average distance invaded plotted against the number of cells invaded. Non-parametric Spearman’s correlation was estimated to evaluate if there was linear association (Spearman’s *r* = 0.63, with *P*-value = 0.009). **(L)** Example of the invasion analysis and centroid tracking. Blue stars are identified cells, and red circles are cells that are counted as invaded cells. Average distance is calculated as distance the cell has deviated from the monolayer.

To investigate cellular invasion rates, confocal z-stack images of intact hydrogels were taken at four fields of view in four biological replicates and analyzed. PAVECs and PAVICs were stained individually with CellTrace™ Far Red and CellTrace™ CSFE, respectively, before seeding onto and into hydrogels. After 2 weeks, hydrogels were imaged, and PAVECs that had deviated from the seeded monolayer were visualized (Figures 4A–D) and quantified using a MATLAB script. Examples of MATLAB centroid tracking for each condition were shown (Figures 4E–H) with an example of the invasion analysis (Figure 4L). The average number of invaded cells was significantly higher for the 2.2 mg/mL collagen (stiffness control) and HA conditions when compared to cells within control hydrogels (1.5 mg/mL collagen) (Figure 4I). Similarly, the average distance invaded was significantly greater for 2.2 mg/mL collagen and HA conditions, and there was an insignificant increase in average distance invaded for the CS conditions when compared to the 1.5 mg/mL collagen control (Figure 4J). Overall, conditions with GAGs promoted greater invasion rates. There was a positive correlation (Spearman’s *r* = 0.63, *P*-value = 0.009) between the number of cells that invaded and the average distance they traveled (Figure 4K).

### Proliferation

**FIGURE 5:**
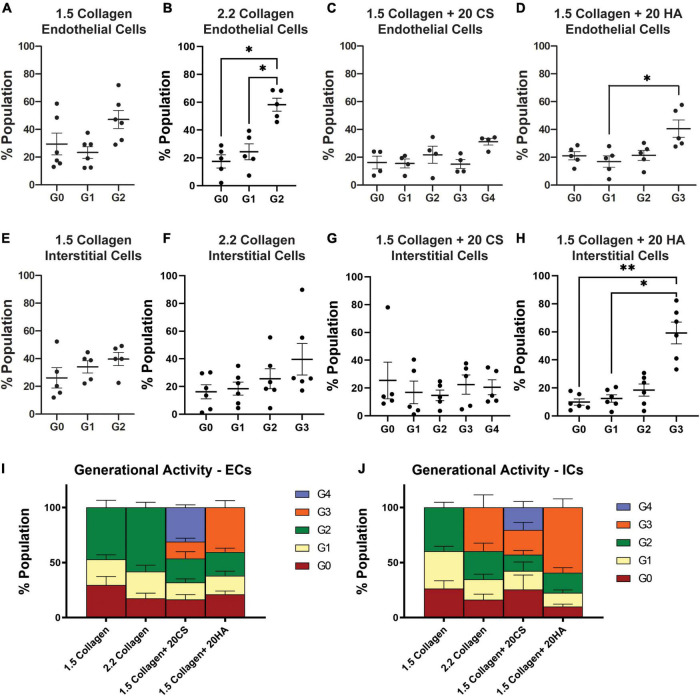
Glycosaminoglycans (GAGs) conditions promote higher levels of cellular proliferation in endothelial and interstitial cells. Interstitial cells were stained with CellTrace CSFE-Green and endothelial cells with CellTrace Far-Red prior to hydrogel seeding. Flow cytometry processing (BC FACS Aria II) was used to measure fluorescence of cells extracted from digested hydrogels after 14 days of growth. FlowJo’s proliferation toolbox was used for analysis. **(A–D)** Generational activity for endothelial cells stained with CellTrace Far-Red across four conditions. **(E–H)** Generational activity for interstitial cells stained with CellTrace CSFE across four conditions. **(I)** Population percentages of each generation and each condition for endothelial cells. **(J)** Population percentages of each generation and each condition for interstitial cells. Data represented as mean ± SEM. *p < 0.05 and **p < 0.01.

Flow cytometry analysis of CellTrace™-stained cells was used to assess *in vitro* proliferation activity in the model. CellTrace™ tracking dyes halve in fluorescence intensity with each cellular division, and the relative fluorescence of each cell measured *via* flow cytometry can provide insight into which generation each cell belongs to. Flow cytometry analysis demonstrated an increase in the number of cellular divisions, represented as a generation number (G0 = initial generation, increasing to as much as G4), in samples with CS (Figures 5C,H) and HA (Figures 5D,I) when compared to 1.5 mg/mL (Figures 5A,F) and 2.2 mg/mL (Figures 5B,G) collagen controls for both PAVECs and PAVICs. Specifically, CS induced the greatest proliferation activity (G4) in both PAVECs and PAVICs (Figures 5E,J).

## Discussion

This study developed an *in vitro* 3D collagen hydrogel model of the aortic valve fibrosa, simulating late-stage CAVD progression in the fibrosa layer by incorporating GAGs into 3D collagen I hydrogels. The model was used to study enzyme activity, cellular proliferation, protein expression, and calcific nodule formation of the resident cell populations. Two GAGs, CS and HA, were incorporated to investigate the role of EndMT-derived aVIC activity in matrix mineralization, and these GAG conditions were found to alter cellular behavior in addition to hydrogel stiffness without the addition of osteogenic medium.

One limitation of the current study is that sex was not addressed. Cells were isolated from female or castrated male pigs. Previous work has shown that there are sex-related differences in gene expression, matrix remodeling, angiogenesis, and early osteogenic markers in porcine and rat aortic valve interstitial cells (36–40). Additionally, previous work has shown that cardiac fibroblasts can be isolated from rats or mice and maintained for a longer period in a quiescent state by decreasing the stiffness of the culture matrix and limiting the nutrient content of the cell culture medium to 2% serum (41), or porcine VIC myofibroblast differentiation can be blocked by culturing with fibroblast growth factor (FGF-2) (42). The control medium used in the current study contained 10% serum without FGF-2, and this alone may have contributed to valve cell differentiation toward myofibroblasts. CS and HA both increased α-SMA expression (Figures 2, 3), invasion rates (Figure 4), and proliferation activity (Figure 5). Additionally, CS conditions yielded a higher level of calcific nodule formation (Figure 1), suggesting that alterations in ECM composition are leading to pathological matrix remodeling. Similar ECM alterations were seen in developmental conditions, but these environments lack disruptive matrix mineralization (20, 43). This model also supplements the understanding of the relationship between changing ECM composition and cellular behavior, including eventual pathological consequences. A better understanding of how ECM composition affects cellular behavior could improve tissue engineered heart valves. Understanding the fate of EndMT-derived aVICs could help to direct interstitial cells toward matrix regeneration/reconstruction, for example, or EndMT could provide a viable drug target for CAVD treatment.

This work indicated that HA does not stimulate either early or late-stage disease (Figure 1). However, Stephens et al. discovered the presence of HA local to calcific nodules in diseased human valves, indicating a potentially upregulating effect of HA on calcific nodule formation (16). Baugh et al. (44) found that HA increased calcification in rat interstitial cells whereas Ohri et al. (45) demonstrated HA to have a hampering effect on matrix mineralization in glutaraldehyde-fixed bovine pericardium. Masters et al. revealed that HA increased porcine valvular interstitial cell activity and ECM production, indicating that HA plays an important role in cardiac morphogenesis and remodeling (27, 46). Alternatively, Porras et al. demonstrated that pathological concentrations of HA and CS were not sufficient to cause *in vitro* disease progression in porcine VICs (29). Although there are discrepancies in the effect that HA has on calcification and disease progression, HA appears to have an overall regulatory effect on valve function. These results confirmed that HA did not exacerbate late-stage mineralization, but did induce significant alterations in cellular behavior (increased proliferation, α-SMA expression, and invasion rates), when compared to collagen-only controls. Lei et al. found that the incorporation of HA into tissue engineered scaffolds seeded with porcine cells promoted cell-mediated tissue remodeling, increased matrix density and stiffness, and regulated tissue contraction (47). Similarly, the current study provides evidence that HA may play a role in matrix regeneration and could be a potential tool for directing tissue activity toward non-pathological matrix remodeling. Recapitulating the role of HA in valvulogenesis could yield greater insight into improved methods for scaffolding and selection of biomaterials for novel tissue engineered valves (47, 48).

In contrast to HA, CS conditions were shown to induce late-stage mineralization (Figure 1), significant α-SMA expression (Figure 2), and increased proliferation rates. This suggests a possible cascade of events that resulted from an increase in EndMT-derived aVIC, which have higher expression of α-SMA (22). Dahal et al. also showed that CS increased pro-calcific markers (myofibroblastic and osteoblastic gene expression) and collagen I production by porcine aVICs (22). Additionally, Mendoza et al. demonstrated that 20 mg/mL CS contributed to CAVD progression in a microfluidic 3D model using PAVIC and PAVEC (35). Interestingly, HA was also shown to increase EndMT-related activity and proliferation levels, but to a lesser extent than CS, and did not result in the same late-stage disease as CS did within the *in vitro* model. Porras et al. also found that CS was effective in retaining lipoproteins classical to early disease markers in a model containing porcine cells, indicating that CS alone may promote a fibrocalcific response (29). Understanding the differences and relationship between these two GAG types could provide guidance in designing engineered scaffolds or disease models. For example, Lei et al. used HA and CS to supplement bioprosthetic heart valve constructs, and concluded that when used together, these GAGs decreased calcification when compared to scaffolds without GAGs (49). This information can contribute to enhanced fabrication of bioprosthetic valves, and can aid in choosing appropriate cell sources for enhanced valvular matrix regeneration properties and the prevention of disease conditions. Further work can focus on the integration of both HA and CS into the 3D model, thereby exploring their relationship.

## Conclusion

We have developed a 3D cell culture model of CAVD by incorporating GAGs into a collagen I hydrogel seeded with porcine aortic valve cells. Findings suggest that the altered ECM containing CS may yield matrix mineralization *via* EndMT-mediated aVICs. In conditions containing HA, matrix mineralization was not present, despite increased levels of EndMT when compared to controls (increased α-SMA expression and cellular invasion). The role of EndMT in valvulogenesis is well-studied, and EndMT is also associated with adult diseases, including cancer (50). The role of EndMT in CAVD disease progression still remains unknown, and this model contributes to the knowledge gap. Leveraging non-calcific EndMT as a tool for directing matrix reconstruction and using natural biomaterials, such as HA and CS, could significantly benefit the production of bioengineered valves. Specifically, this work demonstrates that HA supplementation could be a viable option for long-term scaffolding. Further, this model could also provide a platform for testing new pharmaceutical treatments of late-stage CAVD.

## Data availability statement

The raw data supporting the conclusions of this article will be made available by the authors, without undue reservation.

## Author contributions

BM, PH, M-HC, and GM secured the funding. JB, BA, and MM performed the experiments and collected the data. JB, BA, and M-HC conducted the formal data analysis. JB and BA wrote the manuscript. MM, BM, PH, M-HC, and GM reviewed and edited the manuscript. All authors contributed to the article and approved the submitted version.
